# Household water treatment practice and associated factors in Ethiopia: A systematic review and meta-analysis

**DOI:** 10.1371/journal.pone.0285794

**Published:** 2023-06-08

**Authors:** Belay Desye, Amensisa Hailu Tesfaye, Gete Berihun, Tadesse Sisay, Chala Daba, Leykun Berhanu

**Affiliations:** 1 Department of Environmental Health, College of Medicine and Health Sciences, Wollo University, Dessie, Ethiopia; 2 Department of Public Health, College of Medicine and Health Sciences, Adigrat University, Adigrat, Ethiopia; 3 Department of Environmental and Occupational Health and Safety, Institute of Public Health, College of Medicine and Health Sciences, University of Gondar, Gondar, Ethiopia; Gadjah Mada University Faculty of Medicine, Public Health, and Nursing: Universitas Gadjah Mada Fakultas Kedokteran Kesehatan Masyarakat dan Keperawatan, INDONESIA

## Abstract

The provision of potable water is crucial to ensuring the health and dignity of individuals. In many developing countries, including Ethiopia, waterborne disease has become a major public health problem. There is a significant gap in accessing comprehensive national-wide evidence on Household Water Treatment (HWT) practices and associated factors in Ethiopia. Therefore, this study aims to assess the pooled HWT practice and associated factors in Ethiopia. A comprehensive search of published studies before October 15, 2022, was identified using databases and other sources. Data were extracted using Microsoft Excel, and analysis was performed using STATA 14/SE software. A random-effects model was used to estimate the pooled proportion of HWT practices and the odds ratio of associated factors. The funnel plot and Egger’s regression test were used to assess publication bias, and I^*2*^ test statistics was used to assess heterogeneity. Duval and Tweedie’s "trim and fill" method was performed to adjust the pooled estimate. A subgroup analysis was also conducted to identify the sources of heterogeneity. In this study, a total of 708 articles were retrieved, and 16 eligible studies were included. The pooled proportion of HWT practice in Ethiopia was found to be 21% (95% CI: 17–24). Having a formal education (OR: 2.42, 95% CI (2.11–2.74)), being male (OR: 1.32, 95% CI (1.13–1.51)), owning radio (OR: 1.33, 95% CI (1.18–1.47)), having a higher income (OR: 1.73, 95% CI (1.41–2.04)), unimproved water source (OR: 1.71, 95% CI (1.41–2.01)), fetching water at more frequently (OR: 3.31, 95% CI (1.99–4.64)), dipping methods of water drawing (OR: 2.08, 95% CI (1.66–2.51)), and taken training of water treatment (OR: 2.15, 95% CI (1.55–2.75)) were all found to be associated with HWT practice. Based on the findings of this study, the pooled proportion of HWT practice in Ethiopia was found to be one-fifth, which indicated that it was significantly low. Therefore, the authors recommend that households could better receive adequate information about HWT practices through strengthened health education and intensive training on HWT.

## Introduction

The provision of and access to potable water are crucial to ensuring the health and dignity of individuals [1]. The United Nations recognizes access to safe water and sanitation as a fundamental human right. Everyone has the right to access sufficient, safe, acceptable, affordable, accessible, and continuous water for personal and domestic use [2]. Household water treatment is an important public health intervention as it can be adopted at the point of use in homes, thus minimizing the risk of recontamination. It is especially important when the water source is unimproved and when there is a possibility of recontamination during transport, storage, and consumption processes. Moreover, it can be used even in areas with piped water supply as water supply interruptions happen [1, 3, 4]. Household water treatment has been the most simple and cost-effective means of improving water quality and preventing waterborne diseases [1, 5, 6].

Household water treatment becomes the most appropriate water treatment when communities lack the capacity to develop large-scale water treatment systems due to their many operation and maintenance issues requirements [1]. The major methods of HWT are boiling, filtration, solar disinfection (SODIS), chlorination, and household water storage. Selecting the most appropriate treatment method depends on the condition of water quality, cultural acceptability, feasibility, availability of technology, and other conditions [7, 8].

A significant proportion of diarrheal diseases can be prevented through safe drinking water, adequate sanitation, and hygiene [5, 9]. According to the World Health Organization (WHO), HWT can reduce episodes of diarrheal disease by 39% [7]. Considering this, Sustainable Development Goal (SDG) #6 calls to ensure the availability and sustainable management of water and sanitation for all by 2030 [10]. Globally, safely managed drinking water at home was at 74%, in Sub-Saharan Africa it was at 30%, and in Ethiopia it was at 13% in 2020 [2]. Despite progress towards household coverage of properly safely managed drinking water was slightly improved, 844 million people in the world still lack access to basic water services, and over 2.1 billion people lack access to safely managed drinking water on their premises [2]. Evidence from the Demographic and Health Survey (DHS) revealed that only 18% of households in Sub-Saharan Africa [11] and only 7% of households in Ethiopia appropriately treat their drinking water effectively [12].

Providing safe water to over one billion people who still lack access and millions more who still suffer from contamination of their improved water sources has been recognized as having economic benefits and sustainable health improvements [1, 13]. In most of the developing countries in the world, including Ethiopia, waterborne disease has been becoming a major public health problem due to the consumption of unsafe drinking water [14]. The quality of drinking water being supplied is often neglected, even if access to water supply has been substantially increased. Drinking water supplied by centralized treatment systems is likely to be contaminated due to poor distribution networks, inadequate management, and unhygienic handling prior to consumption [1].

A wide range of studies indicate that HWT can improve drinking water quality prior to consumption and has been found to be an appropriate and acceptable method that can reduce the risk of diarrhea significantly [15–18]. Studies revealed that HWT practice in Ethiopia was found to be in the range of 2.8% [19] to 76.3% [20], and determinant factors like educational status, training, and the nature of water sources were identified [21–23]. The findings were inconsistent, there was significant variation in the reporting level of HWT practice, and they did not have the same statistical significance for implementing appropriate interventions.

Based on the search of the database, there is no national-wide and systematic review and meta-analysis study conducted on the HWT practice and associated factors in Ethiopia. According to the findings, there is inconsistency between the existing evidence, and there is a significant gap in accessing a comprehensive document regarding the HWT practice and associated factors in Ethiopia. Therefore, this review can provide well-organized data on the HWT practice and associated factors in Ethiopia. "What is the proportion of HWT practice in Ethiopia?" and "What are the factors associated with HWT practice in Ethiopia?" were the research questions for this study. The findings of this study could help health authorities, policymakers, Non-Governmental Organizations (NGOs), and WHO to develop and implement an appropriate strategy and effective intervention in HWT practice.

## Materials and methods

### Study setting

This study was conducted in Ethiopia. Ethiopia is located in the northeastern part of Africa, also known as the Horn of Africa. It shares borders with Djibouti to the northeast, Eritrea to the north, Kenya to the south, Somalia to the east and northeast, Sudan to the northwest, and South Sudan to the west. Currently, the Ethiopian population is estimated at 123,415,729, which makes it the second most populous country in Africa after Nigeria [24].

### Protocol and registration

The protocol for this review was registered in the International Prospective Register of Systematic Reviews (PROSPERO), the University of York Centre for Reviews and Dissemination (record code: CRD42022364976, October 17^th^, 2022).

### Information sources and search strategies

This review and meta-analysis were conducted according to the guidelines of Preferred Reporting Items for Systematic Reviews and Meta-Analyses (PRISMA). The four phases drawn from the PRISMA flowchart were documented in the results to show the study selection process from identification to the included studies [25] **(S1 Table)**.

The following databases: PubMed/MEDLINE, Cochrane Library, Science Direct, and AJOL were searched for published studies before October 15, 2022. For the PubMed/MEDLINE search, the following key terms were used in combination with the Boolean operators "AND" and "OR". ("Household Water Treatment" [All Fields] OR "Point-of-Use Water Treatment" [All Fields] OR "Small Scale Water Treatment" [All Fields] OR "Household Water Treatment Practice" [All Fields] OR "Household Water Treatment Technologies" [All Fields] OR "Household Water Treatment Methods" [All Fields] OR "Household Water Treatment and Safe Storage" [All Fields] OR "Household Water Treatment Systems" [All Fields]) AND ("Associated Factors" [All Fields] OR "Determinants"[All Fields] OR "Risk Factors"[All Fields]) AND "Ethiopia" [All Fields].

In addition to the electronic database search, grey literature was identified from Google Scholar and direct Google searches. Moreover, the reference lists (bibliographies) of the included articles were also searched to obtain additional articles.

### Eligibility criteria

This systematic review and meta-analysis included studies conducted in Ethiopia that examined the proportion of HWT practice and associated factors.

#### Inclusion criteria

Articles that met the following criteria were considered for inclusion in this study.

◾ Population: Household heads.
◾ Outcomes of interest: Articles reported the proportion of HWT practice and its associated factors with HWT practice.
◾ Study design: A cross-sectional study.
◾ Study setting: Studies are conducted only in Ethiopia.
◾ Language of published articles: English.
◾ Publication issue: Peer-reviewed journal articles published before October 15, 2022.

#### Exclusion criteria

Systematic reviews, short communications, letters to editors, commentaries, and qualitative studies were excluded. In addition, articles that were not fully accessible after three email contacts with the corresponding author were excluded.

### Study selection process

Two investigators (BD and AHT) independently screened articles by their title, abstract, and full text to identify eligible articles using predetermined inclusion and exclusion criteria. The screened articles were compiled together by two investigators (BD and AHT), and discrepancies were resolved by consensus by involving a third reviewer (GB).

### Data extraction and management

**Fig 1:**
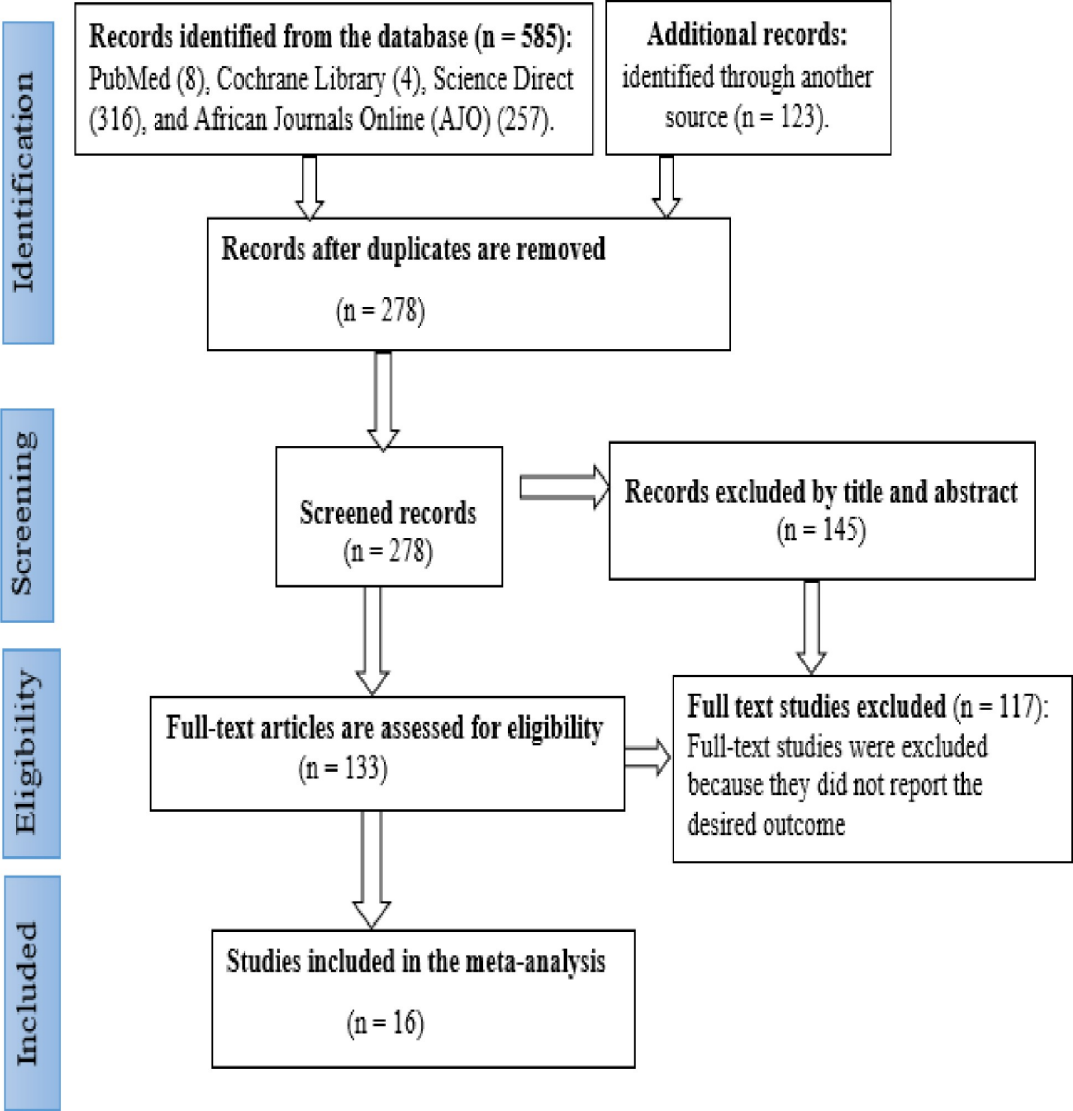
Flow diagram of study selection for this systematic review and meta-analysis, 2023.

The data extraction format included information such as the name of the author and publication year, study area, region, study setting, method of data collection, sampling methods, sample size, response rate, proportion of HWT practice, and risk of bias **(Table 1)**. Zotero reference manager software was utilized to collect and organize search outcomes and for the removal of duplicate articles. The PRISMA flow diagram was used to summarize the selection process **(Fig 1)**.

**Table 1:** Descriptive summary of included studies of HWT practice and associated factors in Ethiopia, 2023.

Author, publication year	Study area	Study region	Study setting	Methods of data collection	Sampling methods	Sample size	Response Rate (%)	Proportion of HWT practice (%)	Risk of bias
Berhanu and Hailu, 2015 [28]	Bona	SNNPR	Rural	IA and SSC	SRS	604	100	26.5	Low
Tafesse et al., 2021 [29]	Gibe	SNNPR	Rural & Urban	IA	STRS	633	99	34.5	Low
Kassie and Hayelom, 2017 [19]	Farta	Amhara	Rural	IA and OC	SRS	834	-	2.8	Moderate
Birara et al., 2018 [20]	Bahir Dar city	Amhara	Urban	IA	Srs	459	91	76.3	Moderate
W/tsaddik et al., 2022 [30]	Bule	SNNPR	Urban	IA	SRS	418	100	29.9	Low
Damtew and Geremew, 2020 [31]	Ethiopia	All-region	Rural & Urban	IA	SRS	16650	-	6.24	Low
Eticha et al., 2022 [21]	Ameya	Oromia	Rural & Urban	IA	SRS	413	-	30.3	Low
Geremew et al., 2018 [22]	Ethiopia	All-region	Rural & Urban	IA	SRS	16650	-	6.5	Low
Admasie et al., 2022 [32]	Sodo Zuria	SNNPR	Rural	IA	SRS	836	99.6	44.1	Low
Belay et al., 2016 [23]	Burie Zuria	Amhara	Rural	IA	SRS	797	94.2	44.8	Low
Bitew et al., 2017 [33]	Dabat	Amhara	Rural & Urban	IA	Srs	845	100	23.1	Low
Anley et al., 2020 [34]	Degadamot	Amhara	Rural	IA	SRS	845	100	14.1	Low
Geremew et al., 2019 [35]	Kersa and Eastern hararge	Oromia and Harari	Rural& Urban	IA	SRS	377	100%	31	Low
Azage et al., 2021 [36]	Baso Liben, Yilmana Densa and Fogera	Amhara	Rural	IA	SRS	865	99.1	6	Low
Merga et al., 2022 [37]	Assosa district	Benishangul	Rural & Urban	IA	SRS	378	95.17	13.2	Moderate
		Gumuz							
Tsegaye et al., 2020 [38]	Degadamot district	Amhara	Rural	IA	SRS	845	100	14	Moderate

**Keys**: SNNPR = South Nations, Nationalities, and people’s representative, IA = Interviewer Administered, SSC = Sanitary Survey Checklist, OC = Observational Checklist, SRS = Systematic Random Sampling, Srs = Simple random sampling, STRS = Stratified random sampling, and— = Not found

### Quality assessment

The Joanna Briggs Institute’s (JBI) quality appraisal tools for cross-sectional studies were used to assess the quality of the included articles and the risk of bias in each study [26]. Two reviewers (BD and AHT) independently assessed the quality of the included articles. The assessment tool contains eight criteria: (1) clear inclusion and exclusion criteria; (2) description of the study subject and study setting; (3) use of a valid and reliable method to measure the exposure; (4) standard criteria used for measurement of the condition; (5) identification of confounding factors; (6) development of strategies to deal with confounding factors; (7) use of a valid and reliable method to measure the outcomes; and (8) use of appropriate statistical analysis. It was evaluated using the JBI critical appraisal checklist of cross-sectional study options: yes, no, unclear, and not applicable. The risks for biases were classified as low (total score, 6 to 8), moderate (total score, 3 or 5), or high (total score, 0 to 2). Finally, articles with low and moderate biases were considered in this review **(S2 Table)**.

### Outcome of interest

There are two main outcomes of this review. The primary outcome of this study was the pooled proportion of HWT practice. It was determined using a percentage (%). The second outcome of this review was the pooled measure of the association between the HWT practice and associated factors in Ethiopia. It was determined using the pooled odds ratio (OR) with a 95% confidence interval.

### Statistical methods and data analysis

The extracted data were exported from a Microsoft Excel spreadsheet to STATA version 14/SE for further analysis. Heterogeneity among the included studies was quantitatively measured by the index of heterogeneity (*I*^*2*^ statistics), in which 25%–50%, 50%–75%, and >75% represented low, moderate, and high heterogeneity, respectively [27]. The overall pooled estimate was computed using the metaprop STATA command using the DerSimonian–Laird random effect model. Subgroup analysis was done by region, study setting, and year of publication to see the difference in the pooled proportion of HWT practices. The small-study effect was evaluated using the funnel plot and Egger’s regression test, with a p-value ≤ 0.05 as a cutoff point to declare the presence of publication bias. A p-value ≤ 0.05 was used to declare the association as statistically significant at a 95% confidence level. The results were presented using graphs, tables, texts, and a forest plot.

## Results

### Searching process

Using the database and manual searching, a total of 708 articles were retrieved. After the duplication was removed, there were 278 articles remaining. Based on their titles and abstracts, 145 articles were excluded. In addition, 117 articles were excluded because they did not report the outcome of interest. Finally, 16 articles were included in this study **(Fig 1)**.

### Characteristics of the included studies

The characteristics of the review, which include the publication year, study location, study region, study setting, methods of data collection, sampling methods, sample size, response rate, and proportion of HWT practice, are all compiled in **(Table 1)**. By design, all included studies were cross-sectional. This study included 16 studies with a total of 42,449 participants [19–23, 28–38]. The included articles were conducted between 2015–2022. The included studies had an average response rate of 98.5%. The included study sample sizes ranged from 377 to 16, 650. In this review, a study conducted in the Amhara region at the Farta study site had the lowest proportion of HWT practice (2.8%), while a study conducted in the Amhara region at the Bahir Dar city study site had the highest proportion of HWT practice (76.3%). The majority of the included studies were conducted using interviewers to administer questionnaires. Seven studies from the Amhara region [19, 20, 23, 33, 34, 36, 38]; four studies from the SNNP region [28–30, 32]; one study from the Oromia region [21]; one study from the Benishangul-Gumuz region [37]; one study from both the Oromia and Harari regions [35]; and two studies from the all-region [22, 31] were used to obtain the pooled HWT practices and associated factors **(Table 1)**.

### Meta-analysis

#### Pooled proportion of HWT practice in Ethiopia

**Fig 2:**
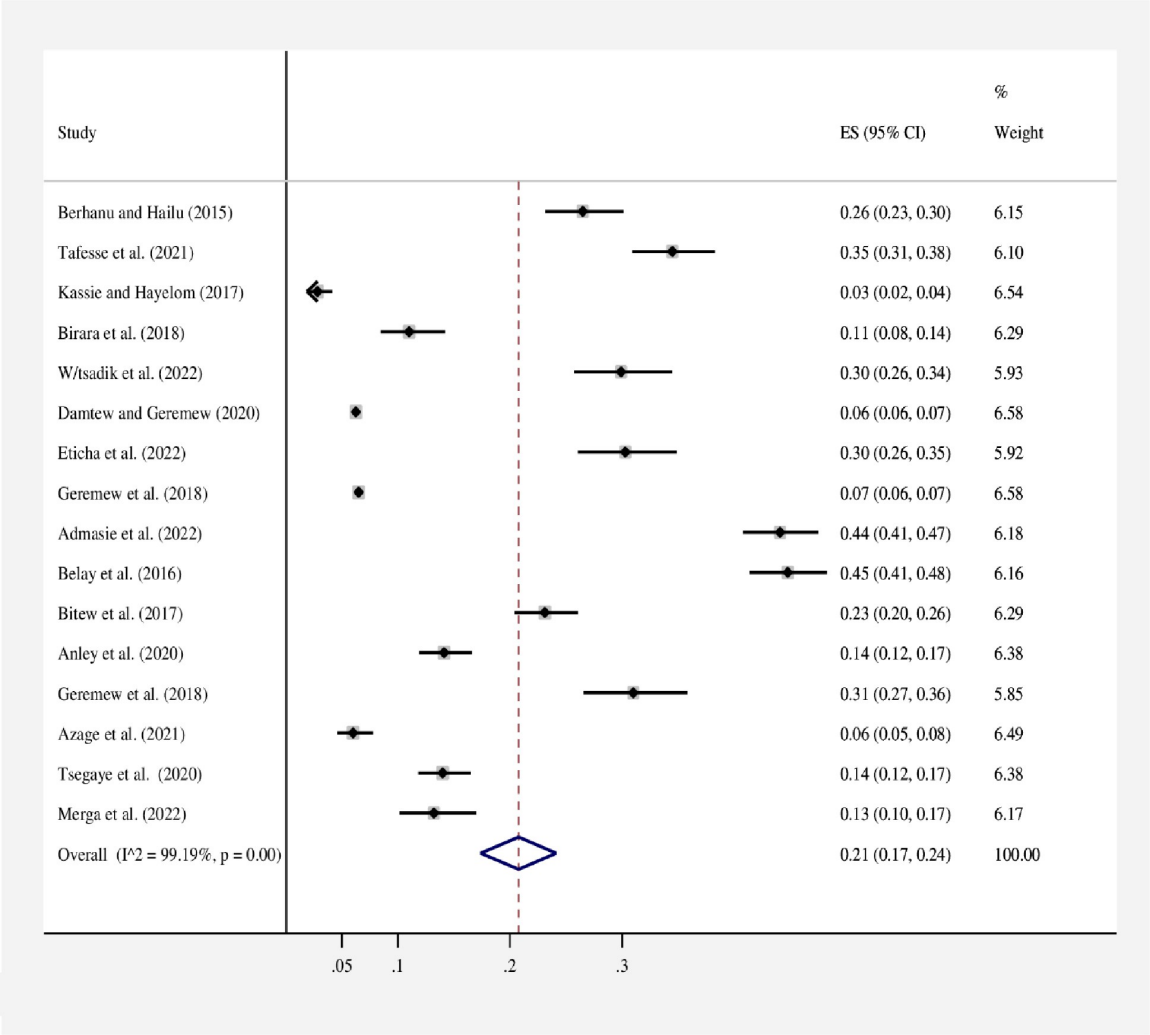
Forest plot of the proportion of HWT practices in Ethiopia, 2023. Note: Weights are from random effects model.

The proportion estimate varied among the included studies with significant heterogeneity (*I*^*2*^ = 99.19%, p = 0.00). According to the random effects model, the pooled proportion of HWT practice was 21% (95% CI: 17–24). In this meta-analysis, the proportion of HWT practices in Ethiopia ranged from 3% (95% CI: 2–4) in the Farta study area of the Amhara region [19] to 45% (95% CI: 41–48) in the Burie Zuria of the Amhara region. A forest plot depicts the proportion estimates of HWT practice in Ethiopia **(Fig 2)**.

#### Subgroup analysis

**Fig 3:**
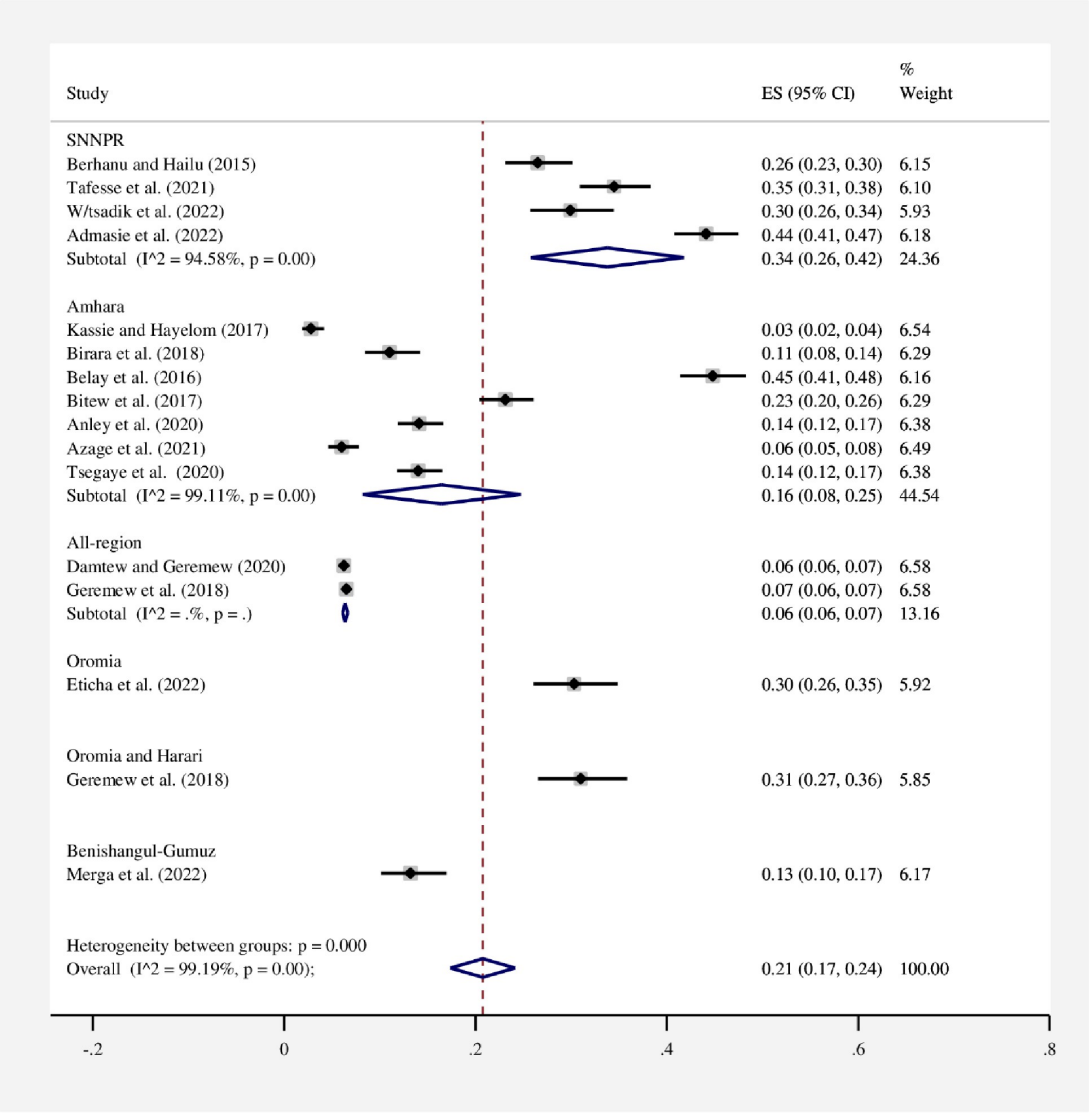
Subgroup analysis by region for the pooled proportion of HWT practice in Ethiopia, 2023. Note: Weights are from random-effects model.

**Fig 4:**
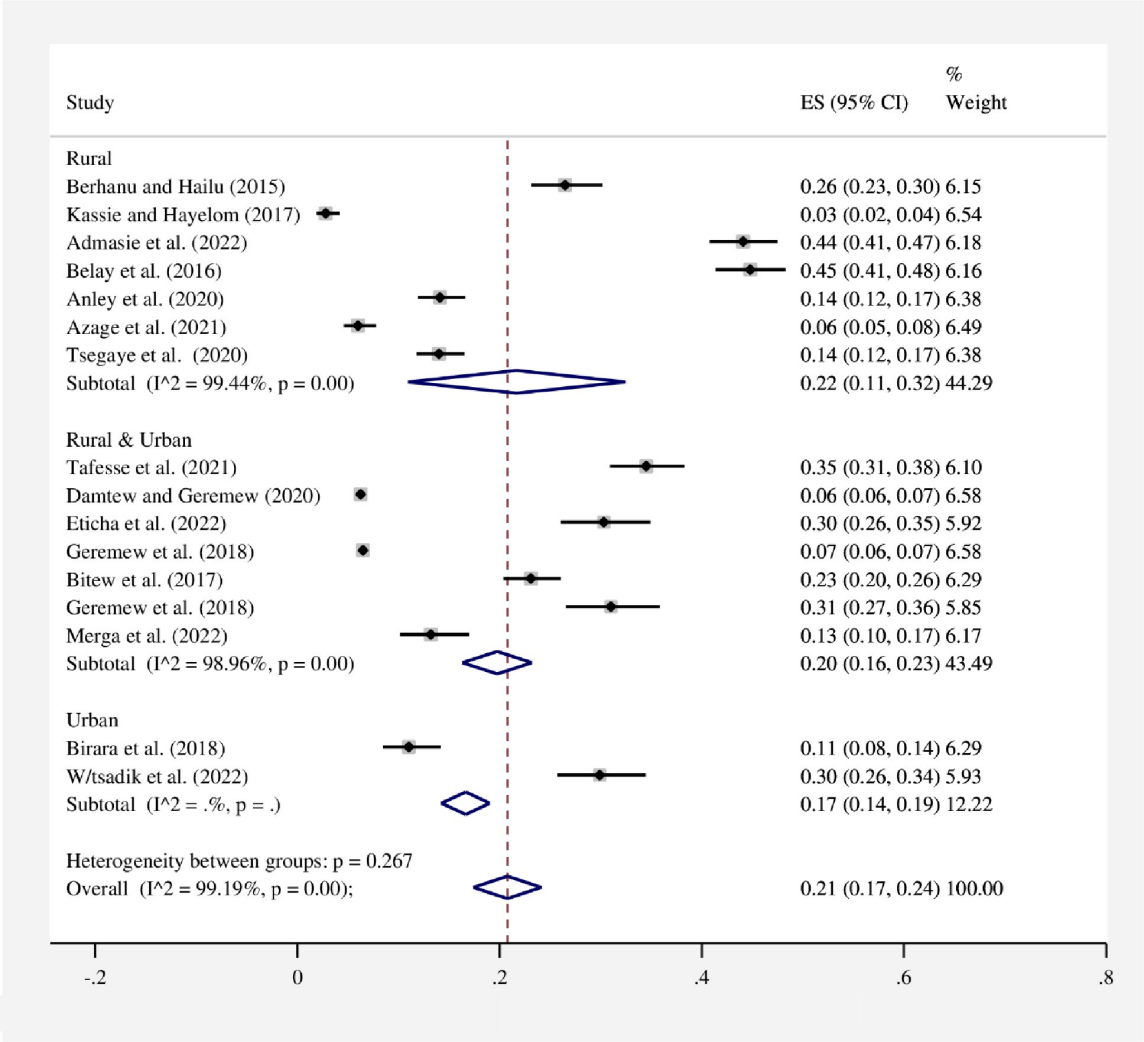
Subgroup analysis by study settings for the pooled proportion of HWT practice in Ethiopia, 2023. Note: Weights are from random-effects model.

**Fig 5:**
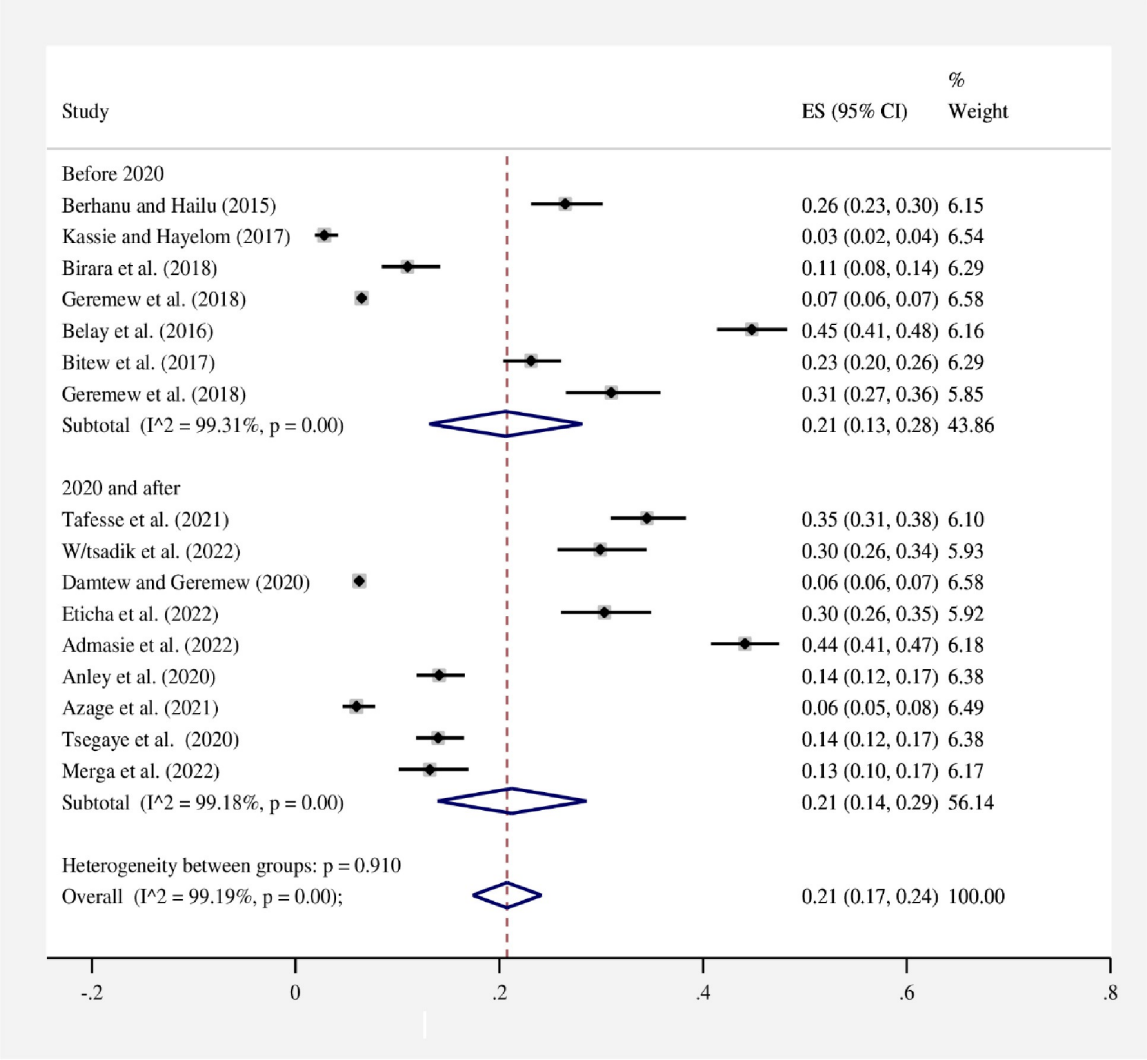
Subgroup analysis by year of publication for the pooled proportion of HWT practice in Ethiopia, 2023. Note: Weights are from random-effects model.

In this study, to perform subgroup analysis, a study region, study setting, and year of publication were used **(Figs 3–5)**. As a result, the study’s subgroup analysis revealed that in the SNNPR region, 34% (95% CI: 26–42) and in all regions, 6% (95% CI: 6–7) had the highest and lowest pooled proportions of HWT practice, respectively. Subgroup analysis based on study settings of rural, rural and urban, and urban of the pooled HWT practice was found to be 22% (95% CI: 11–32), 20% (95% CI: 16–23), and 17% (95% CI: 14–19), respectively. On the other hand, a subgroup analysis based on the year of publication was also conducted to see if there were any year-to-year differences in HWT practice. As a result, 21% (95% CI: 13–28) of the pooled proportion of HWT practice was found before 2020, and similarly, 21% (95% CI: 14–29) of the pooled proportion of HWT practice was found after 2020.

#### Heterogeneity and publication bias

**Fig 6:**
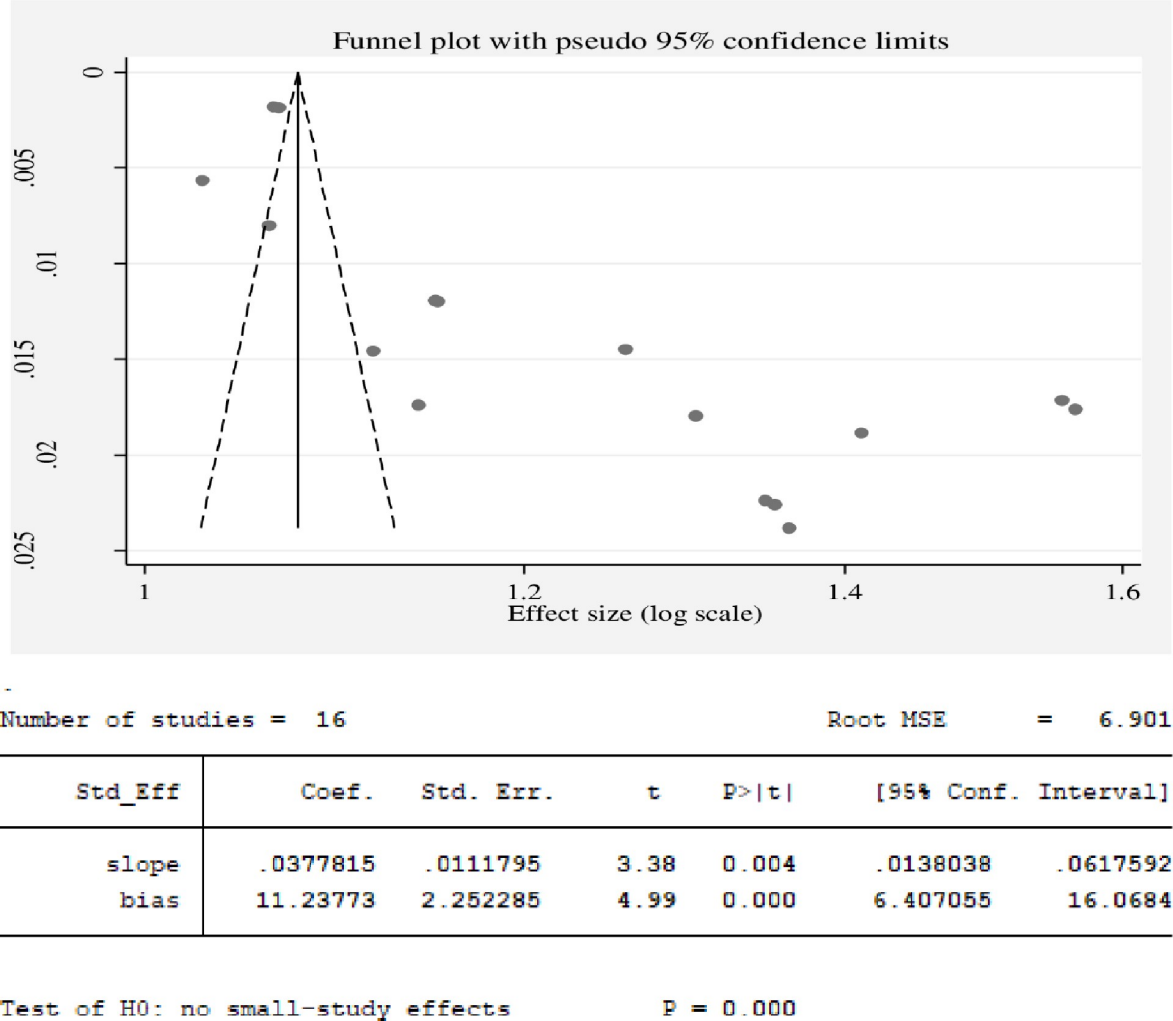
Funnel plot and Egger’s regression test, respectively, studies of the pooled proportion of HWT practice in Ethiopian, 2023.

**Fig 7:**
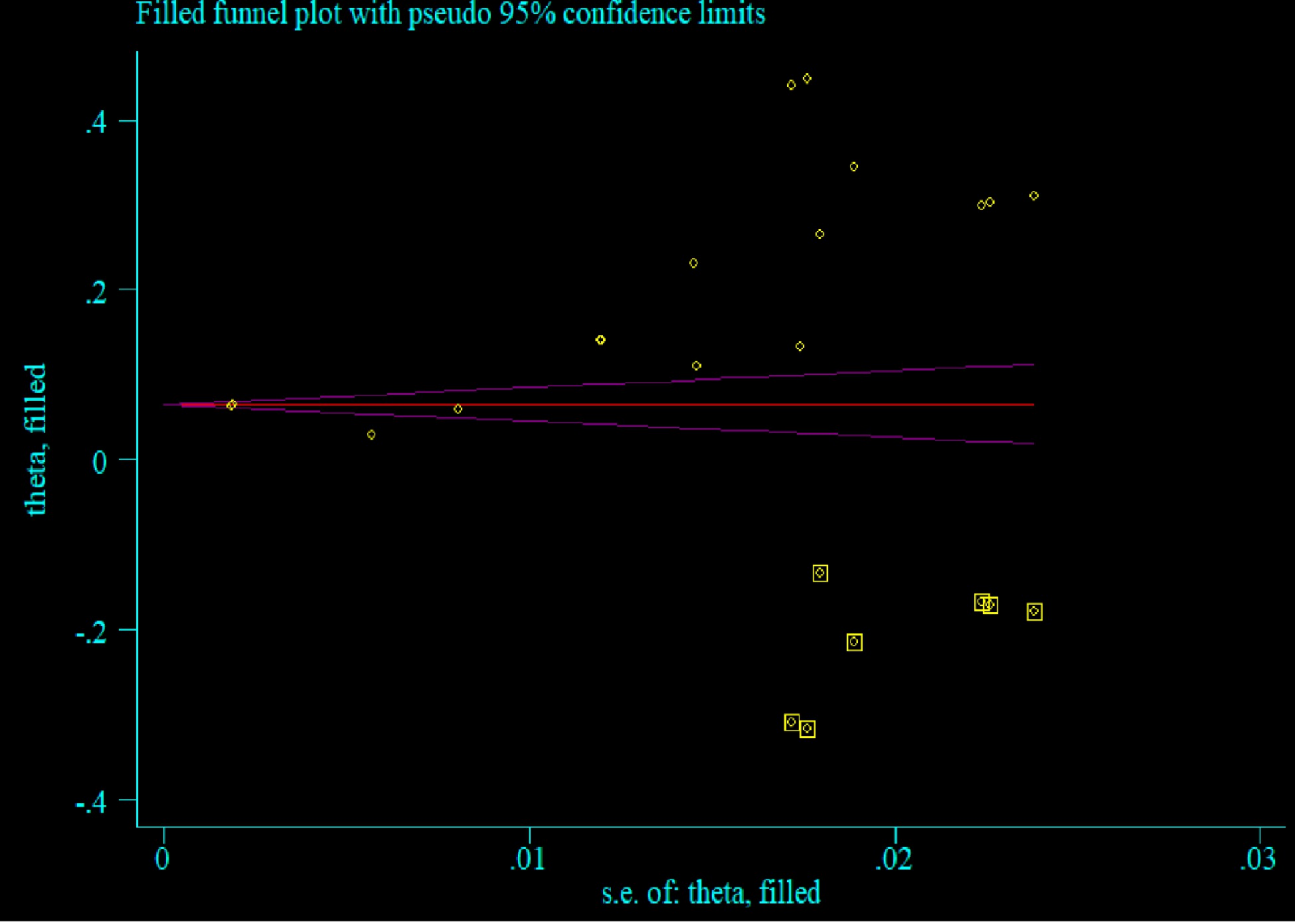
Funnel plot with 95% CI of a simulated meta-analysis containing 16 studies, 2023.

The existence of heterogeneity and publication bias was determined within the included studies. The included studies had a high level of heterogeneity (*I*^2^ = 99.19%, p = 0.00). The presence of possible small study effects was checked by using a funnel plot and Egger’s regression test to declare the presence of publication bias. The funnel plot revealed that the distribution of studies was asymmetrical, whereas Egger’s regression test revealed that estimating the pooled proportion of HWT practice was statistically significant (p = 0.00), which means there is a publication bias **(Fig 6)**. Thus, Duval and Tweedie’s “trim and fill” method was performed to account for publication bias **(Fig 7)**.

#### Factors associated with HWT practices in Ethiopia

In this meta-analysis, factors associated with HWT practice were assessed using 16 studies [19–23, 28–38]. Among the 16 articles, the findings of 9 studies [21–23, 28, 29, 31, 33, 34, 36] revealed that HWT practices were significantly associated with educational status. As a result, the likelihood of HWT practice occurring was 2.42 times higher among households that are having formal education than those that are not (OR: 2.42, 95% CI (2.11–2.74)). Households headed by men were 1.32 times more likely to practice HWT than those headed by women (OR: 1.32, 95% CI (1.13–1.51)).

Similarly, the pooled results of this meta-analysis revealed that households with radio ownership were 1.33 times more likely to practice HWT than those without (OR: 1.33, 95% CI (1.18–1.47)). Households with a better income status were 1.73 times more likely to practice HWT than those with a lower income status, according to the pooled results of this meta-analysis (OR: 1.73, 95% CI (1.41–2.04)). Household heads who received training about water treatment were 2.15 times more likely to practice HWT than those who did not (OR, 2.15, 95% CI (1.55–2.75)).

A meta-analysis of the relationship between the nature of water sources and HWT practices found that households that used unimproved water sources were 1.71 times more likely to practice HWT than those that used improved water sources (OR: 1.71, 95% CI (1.41–2.01)). Households with a higher frequency of fetching water were 3.31 times more likely to practice HWT than those with a lower frequency of fetching water (OR: 3.31, 95% CI (1.99–4.64)). Finally, households that used the dipping method of water drawing are 2.08 times more likely to practice HWT than households that used the pouring method of water drawing (OR: 2.08, 95% CI (1.66–2.51)) **(Table 2)**.

**Table 2:** The pooled effect size of factors associated with HWT in Ethiopia, 2023.

S.N	Variables (Reference)	Authors and *I*^2^ with p-value	Odds ratio (95% CI)
1.	Educational status (Illiterate)	Tafesse et al.	2.01(1.34, 3.00)
		Berhanu and Hailu	3.00(1.50, 6.04)
		Eticha et al.	1.60(1.02, 2.93)
		Geremew et al.	3.01(2.47, 3.66)
		Damtew and Geremew	2.50(1.43, 4.36)
		Belay et al.	2.07(1.51, 2.83)
		Bitew et al.	2.49(2.11, 2.74)
		Azage et al.	1.76(1.02, 3.05)
		Anley et al.	5.81(3.60, 9.38)
		Overall, IV (*I*^2^ = 57.3%, p = 0.016)	**2.42(2.11, 2.74)**
2.	Sex (Female)	Belay et al.	1.80(1.24, 2.62)
		Geremew et al.	1.28(1.28, 1.68)
		Overall, IV (*I*^2^ = 50.3%, p = 0.156)	**1.32(1.13, 1.51)**
3.	Owning radio (No)	Geremew et al.	1.21(1.07, 1.38)
		Eticha et al.	2.80(2.21,3.32)
		Overall, IV (*I*^2^ = 96.6%, p = 0.000)	**1.33(1.18, 1.47)**
4.	Income status (Low)	W/tsadik et al.	2.37(1.22, 4.6)
		Admasie et al.	1.50(1.23, 3.47)
		Geremew et al.	1.55(1.14, 2.11)
		Damtew and Geremew	1.97(1.12, 3.47)
		Bitew et al.	1.78(1.33, 2.37)
		Anley et al.	2.78(1.50, 5.15)
		Overall, IV (*I*^2^ = 0.0%, p = 0.745)	**1.73(1.41, 2.04)**
5.	Water sources (Improved)	Geremew et al.	1.76(1.39, 2.22)
		Bitew et al.	1.52(1.13, 2.04)
		Eticha et al.	2.99(1.97, 4.94)
		Overall, IV (*I*^2^ = 44.1%, p = 0.167)	**1.71(1.41, 2.01)**
6.	Frequency of fetching water (Low)	Tafesse et al.	2.65(1.45, 4.88)
		Admasie et al.	2.80(1.21, 9.17)
		Belay et al.	4.87(3.00, 7.91)
		Overall, IV (*I*^2^ = 8.4%, p = 0.336)	**3.31(1.99, 4.64)**
7.	Methods of water drawing (Pouring)	Tafesse et al.	1.86(1.20, 2.87)
		Admasie et al.	1.67(1.14, 2.42)
		Belay et al.	3.41(2.48, 4.69)
		Anley et al.	2.38(1.52, 3.70)
		Overall, IV (*I*^2^ = 61.0%, p = 0.053)	**2.08(1.66, 2.51)**
8.	Water treatment training (No)	Eticha et al.	2.99(1.97, 4.94)
		Azage et al.	1.99(1.44, 2.75)
		Overall, IV (*I*^2^ = 31.4%, p = 0.227)	**2.15(1.55, 2.75)**

## Discussion

The provision of potable water is crucial to ensuring the health and dignity of individuals [1]. Hence, HWT is one of the possible methods to improve the quality of drinking water, and it can reduce water-related diseases in developing countries like Ethiopia [39]. In this study, the pooled proportion of HWT practice was found to be 21% (95% CI: 17–24). The pooled proportion of HWT practice was found to vary from region to region and even within regions, and there is also variation in the study setting: rural (22%), rural and urban (20%), and urban (17%). In this study, educational status, sex, radio ownership, income status, water source, frequency of fetching water, methods of water drawing, and water treatment training were all found to be associated with HWT practice.

The finding of the pooled proportion of HWT practice (21%) in this study was found to be significantly lower than a study conducted in Nigeria (45%) [40], Kenya (69%) [41], Indonesia (51%) [42], and India (53%) [43]. The possible explanation for the difference might be due to the variation in accessibility of information about HWT, the study period, and the nature of water source coverage among the countries. The low level of HWT practice in this study might be also due to the unavailability of treatment options, socioeconomic conditions, and knowledge or awareness gaps.

The possible plausibility of the variation from region-to-region and within the region might be due to variation in the socio-economic, environmental, and perceptional factors of households as well as the nature of water sources. In the present study, there was a significant degree of heterogeneity among the included studies, which is also supportive evidence for the variation in this study. This heterogeneity might be due to differences in study settings, sample size, study population, and training given to the study population as an intervention [44].

From the findings of the present study, the pooled proportion of HWT practice in a study conducted in rural areas was found to be relatively higher than in urban areas. This is supported by Eticha et al. [21] and Belay et al. [23]. HWT is mostly practiced in areas with unimproved water sources, which are mostly used by rural communities. The possible reason might be due to the risk of potential contamination from unimproved water sources.

In the present study, households with formal education were 2.42 times more likely to practice HWT than those that did not have formal education (OR: 2.42, 95% CI (2.11–2.74)). This finding was supported by a study conducted in Egypt [45] and Indonesia [42]. The possible explanation for this finding might be due to the fact that people who are more educated may have a better understanding of the health risks of drinking contaminated water and might know different types of water treatment methods [42]. Similarly, in this study, households that had taken training about water treatment were found to be 2.15 times more likely to practice HWT than those that had not taken the training. This finding is supported by Komarulzaman et al. [46], they reported that training and education are crucial to ensure HWT practice effectively. Therefore, health care professionals would do better to provide intensive training to households on the HWT, which encourages changes in health behaviour, as it is an appropriate strategy to improve good practice [47–49].

In this study, higher-income households were 1.73 times more likely to practice HWT than lower-income households (OR: 1.73, 95% CI (1.41–2.04)). This finding is supported by a study conducted in low- and middle-income countries [50]. The present finding is also supported by Daniel et al. [51], socioeconomic issues are important factors for HWT adoption. The possible reason behind this might be that households with higher incomes have the ability to afford materials for water treatment than those with lower incomes [33], and households in developing countries like Ethiopia, which focus on fulfilling food requirements for their families, do not focus on treating water at the household level [30].

The present study reported that households that used unimproved water sources, which are not protected from external sources of contamination, were 1.71 times more likely to practice HWT than those that used improved water sources (OR: 1.71, 95% CI (1.41–2.01)). This finding is supported by WHO [1], which explains households with unimproved sources that have a potential risk of contamination. This could be explained by the fact that households that believe their water source is contaminated or unimproved are more likely to try to treat their water at home than those who use improved water sources [23, 33].

This study showed that households who fetched water more frequently were 3.31 times more likely to practice HWT than those households who fetched water less frequently (OR: 3.31, 95% CI (1.99–4.64)). This finding is supported by a study conducted in Nigeria [40]. The possible plausibility could be that those who were fetching water more frequently had a higher likelihood to store their water, allowing them to treat their water by storing it through sedimentation [32]. The finding might imply that access to water and storage containers was crucial for households to implement the ability to practice HWT [42].

This study also revealed that participants who draw their water from a storage container by dipping were 2 times more likely to practice HWT than those who draw their water by pouring (OR: 2.08, 95% CI (1.66–2.51)). This finding is supported by WHO [39] and Tafesse et al. [29]. The possible reason for this result might be the fact that those who withdraw water from the storage container by the dipping method increase the risk of potential contamination. Hands can enter the container and contaminate the water during the withdrawal of water by the dipping method. Therefore, the dipping method is not a safe method of water handling; hence, to avoid those contaminants, households may practice the HWT method [52].

This study has its own limitations. This study was considered a study conducted using a cross-sectional study design, so it could not establish cause-and-effect relationships. This study also considered only articles published in English that were used for this systematic review and meta-analysis. Moreover, this study was not considered a qualitative study that reported the psychological factors or perceptions of people.

## Conclusions

Based on the findings of this study, the pooled proportion of HWT practice in Ethiopia was found to be one-fifth, which indicated that it was significantly low. The major determinant factors contributing to low HWT practice in this study was lack of formal education, low frequency of fetching water, poor hygiene practices, and a lack of adequate information and training. Therefore, the authors recommend that households could better receive adequate information about HWT practices through strengthened health education and intensive training on HWT to improve their HWT practice. Moreover, the concerned body could also better provide households with access to water.

## Supporting information

S1 Table PRISMA-P 2009 checklist. (DOCX)

S2 Table Results of JBI quality assessment. (DOCX)
