# Supplementary material for: Household water treatment practice and associated factors in Ethiopia: A systematic review and meta-analysis
Source: PLoS One. 2023 Jun 8;18(6):e0285794. doi: 10.1371/journal.pone.0285794 (PMC10249828; doi:10.1371/journal.pone.0285794)
Supplement: S2 Table — (DOCX) [file pone.0285794.s002.docx]

**Results of JBI Quality Assessment**

| Studies | Clear eligibility criteria | Description of study subject and study setting | Valid and reliable method to measure the exposure | Standard criteria used for measurement of the condition | Identification of confounding factors | Develop of strategies to deal with confounding factors | Valid and reliable method to measured outcomes | Appropriate statistical analysis | Total score out of 8 | Level of bias |
| --- | --- | --- | --- | --- | --- | --- | --- | --- | --- | --- |
| Berhanu and Hailu | Yes | Yes | Yes | Yes | N/A | Yes | Yes | Yes | 8 | Low |
| Tafesse et al. | No | Yes | Yes | Yes | N/A | Yes | Yes | Yes | 7 | Low |
| Kassie and Hayelom | Yes | Yes | Yes | No | N/A | No | No | No | 4 | Moderate |
| Birara et al. | Unclear | Yes | Unclear | Yes | N/A | Yes | Unclear | Yes | 5 | Moderate |
| W/tsaddik et al. | No | Yes | Yes | Yes | N/A | Yes | Yes | Yes | 7 | Low |
| Merga et al. | Yes | Unclear | Unclear | Unclear | N/A | NO | Unclear | Yes | 3 | Moderate |
| Damtew and Geremew | Unclear | Yes | Yes | Yes | N/A | Yes | Yes | Yes | 7 | Low |
| Tsegaye et al. | No | Unclear | Yes | Unclear | N/A | No | Unclear | Yes | 3 | Moderate |
| Abera et al. | No | Unclear | Unclear | Unclear | N/A | No | No | Unclear | 1 | High |
| Eticha et al. | Unclear | Yes | Yes | Yes | N/A | Yes | Yes | Yes | 7 | Low |
| Geremew et al. | Yes | Yes | Yes | Yes | N/A | Yes | Yes | Yes | 8 | Low |
| Admasie et al. | Unclear | Yes | Yes | Yes | N/A | Yes | Yes | Yes | 7 | Low |
| Belay et al. | Unclear | Yes | Yes | Yes | N/A | Yes | Yes | Yes | 7 | Low |
| Bitew et al. | Unclear | Yes | Yes | Yes | N/A | Yes | Yes | Yes | 7 | Low |
| Anley et al. | Yes | Yes | Yes | Yes | N/A | Yes | Yes | Yes | 8 | Low |
| Usman et al. | No | Yes | Unclear | Unclear | N/A | No | Unclear | No | 2 | High |
| Geremew et al. | Yes | Yes | Unclear | Yes | N/A | Yes | Yes | Yes | 7 | Low |
| Azage et al | Yes | Yes | Unclear | Yes | N/A | Yes | Yes | Yes | 7 | Low |
